# Colectomie laparoscopique versus colectomie par laparotomie dans le traitement des adénocarcinomes coliques non métastatiques

**DOI:** 10.11604/pamj.2016.25.165.10071

**Published:** 2016-11-16

**Authors:** Rached Bayar, Zeineb Mzoughi, Achref Djebbi, Ghassen Halek, Mohamed Taher Khalfallah

**Affiliations:** 1 Université de Tunis El Manar, Faculté de Medecine de Tunis, 1007, Tunis, Tunisie; 2 Service de Chirurgie Viscérale CHU Mongi Slim, Sidi Daoued La Marsa, Tunisie

**Keywords:** Laparoscopie, laparotomie, adénocarcinome, colon, Laparoscopy, laparotomy, adenocarcinoma, colon

## Abstract

**Introduction:**

La colectomie laparoscopique pour cancer colorectal constitue, de plus en plus, le traitement de référence. L’objectif de notre travail est de montrer que les résultats à court terme etla sécurité oncologique que procure la voie laparoscopique sont au moins équivalents à ceux de la laparotomie dans le traitement des adénocarcinomes coliques non métastatiques. Nous nous proposons également d’étudier l’impact de la courbe d’apprentissage sur les résultats de la laparoscopie dans ces cancers.

**Méthodes:**

Il s’agit d’une étude rétrospective incluant tousles patients opérés pour des adénocarcinomes coliques résécablessur une période de 6 ans. La population de l’étude était répartie en 2 groupes, selon la voie d’abord utilisée initialement. Le groupe « OC » comprenait 35 patients opérés par laparotomie médiane et le groupe « LAC » comprenait 30 patients opérés par laparoscopie. Toutes les données étaient analysées au moyen du logiciel SPSS version 19.0.

**Résultats:**

Notre étude n’a pas montré de différence significative dans les résultats à court terme entre les 2 groupes à savoir la morbidité per-opératoire, le séjour hospitalier, le séjour en milieu de réanimation ainsi que la morbi-mortalité postopératoire. Concernant les résultats à long terme, il n’y avait également pas de différence significative en termes de complications tardives, type de récidive, survie globale et survie sans récidive. La sécurité oncologique, attestée par les limites de résections et le nombre de ganglions prélevés, n’était pas significativement différente entre les deux groupes. Le temps opératoire était significativement plus long en laparoscopie (p < 0,001). Le taux de conversion était de 33%. Il est passé de 67% au cours des 2 premières années de l’étude à 13% au cours des 2 dernières années. La conversion de la laparoscopie en laparotomie n’avait pas d’impact significatif ni sur les suites opératoires précoces, ni sur la survie globale et la survie sans récidive.

**Conclusion:**

La voie laparoscopique est une voie d’abord ayant des résultats à court et à long terme au moins équivalents à la laparotomie. La courbe d’apprentissage représentant un « passage obligé » n’a pas d’impact négatif sur les résultats du traitement laparoscopique des cancers coliques non métastatiques.

## Abstract (en)

**Introduction:**

Laparoscopic colectomy is considered with increasing frequency the gold standard treatment for colorectal cancer. Our study aims to show that short-term results and the oncological safety of laparoscopy are at least equivalent to those of laparotomy in the treatment of non-metastatic colic adenocarcinomas. We also highlight the impact of the learning curve on outcomes after laparoscopy in patients with these cancers.

**Methods:**

We conducted a retrospective study of all patients undergoing surgery for resectable colic adenocarcinomas over a period of 6 years. The study population was divided into 2 groups based on the surgical procedure used initially. The group “OC“ included 35 patients who underwent midline laparotomy and the group “LAC” included 30 patients who underwent laparoscopy. All data were analyzed using SPSS software version 19.0.

**Results:**

Our study showed that there was no significant difference in short-term outcomes between the 2 groups, namely intraoperative morbidity, hospital stay, intensive care unit stay as well as postoperative morbidity and mortality. Regarding the long-term outcomes, there was also no significant difference in the incidence of late complications, type of recurrence, overall survival and disease-free survival. Oncological safety based on the limits of resection and the number of lymph nodes removed was not significantly different between the two groups. Operative time was significantly longer in the laparoscopic group (p <0.001). Convertion rate was 33%. It went from 67% in the first 2 years of the study to 13% in the last 2 years. The conversion from laparoscopy to laparotomy had no significant impact neither on early postoperative outcomes nor on overall survival and disease-free survival.

**Conclusion:**

Laparoscopy is a surgical procedure resulting in at least equivalent short and long term outcomes as laparotomy. The learning curve representing a “prerequisite” has no negative impact on the outcomes of laparoscopic treatment of non-metastatic colic cancers.

## Introduction

L’incidence des cancers coliques n’a cessé d’augmenter au fil des années [1]. La prise en charge chirurgicale des cancers colo-rectaux a été profondément modifiée par l’avènement de la laparoscopie [1]. L’objectif de notre travail est de montrerque les résultats à court terme et la sécurité oncologique que procure la voie laparoscopique sont au moins équivalents à ceux de la laparotomie dans le traitement des adénocarcinomes (ADK) coliques nonmétastatiques. Nous nous proposons également d’étudier l’impact de la courbe d’apprentissage sur les résultats de la laparoscopie dans ces cancers.

## Méthodes

Nous avons mené une étude rétrospective, uni-centrique. Cette étude avait colligé 65 patients opérés pour des ADK du colon résécables et non métastatiques. Le travail était mené sur une période de 6 ans allant de Janvier 2006 à Décembre 2011. Les critères d’inclusion étaient: critères histologiques : étaient retenus seulement les patients porteurs d’ADK colique prouvé par l’examen anatomopathologique des fragments biopsiques et/ou de la pièce opératoire; Le siège de la tumeur: situé entre la valvule de Bauhin et la charnière recto-sigmoïdienne; Sujets des 2 sexes dont l’âge et l’état (OMS < 3, ASA = III) permettaient un traitement curatif ; Les ADK coliques classés de T1 à T4a et non métastasiques (M0) selon laclassification TNM 2009 de l’union internationale contre le cancer (UICC).

Les critères de non inclusion étaient: les ADK coliques à localisations multiples; Les formes compliquées d’occlusion, de suppuration, de fistulisation ou d’hémorragie; Les ADK coliques entrant dans le cadre d’une polypose adénomateuse familiale (PAF) avérée ou du syndrome de cancer colorectal héréditaire non polyposique (HNPCC) ou syndrome de Lynch; Les antécédents de chirurgie colique ; L’existence d’une obésité sévère ou morbide avec un indice de masse corporelle (IMC) = 35 kg/m^2^.

Les critères d’exclusion étaient: Les tumeurs localement avancées et/ou métastatiques de découverte per-opératoire; L’intolérance au pneumopéritoine. La population de notre étude était répartie en 2 groupes selon la voie d’abord utilisée initialement. Le 1^er^ groupe opéré par laparotomie était appelé groupe «Open Colectomy» (OC). Le 2^ème^ groupe opéré par laparoscopie était appelé groupe «Laparoscopic-Assisted Colectomy» (LAC).Le choix de la voie d’abord était laissé au chirurgien. Les variables étudiées étaient les données épidémiologiques (âge, sexe, IMC, antécédents), les caractéristiques morphologiques et histologiques des tumeurs, les examens biologiques, radiologiques et endoscopiques, la voie d’abord, durée d’intervention et les gestes opératoires. Les critères de jugement principaux étaient les résultats à court terme (mortalité, morbidité post opératoire, séjour hospitalier) et à long terme (survie globale et survie sans récidive) ainsi que la sécurité oncologique définie par les limites de résection et le nombre de ganglions prélevés. Les comparaisons de 2 moyennes sur séries indépendantes étaient effectuées au moyen du test t de Student pour séries indépendantes. Les comparaisons de pourcentages sur séries indépendantes étaient effectuées par le test du khi-deux de Pearson, et en cas de significativité au test du khi-deux et de non-validité de ce test et de comparaison de 2 pourcentages, par le test exact bilatéral de Fisher. Les données de survie étaient étudiées en établissant des courbes de survie selon la méthode de Kaplan Meier.Le seuil de signification était fixé à 0,05. Toutes les données étaient analysées au moyen du logiciel SPSS version 19.0.

## Résultats

### Population étudiée

Soixante-cinq patients étaient inclus dans notre étude. Trente-cinq patients (54%) ont été opérés par voie classique (OC) et 30 patients (46%) ont eu une laparoscopie (LAC).Le taux de conversion de la laparoscopie en laparotomie étaitde 33% (10 patients/30). La médiane d’âge de nos patients était de 66 ans dans le groupe OC. Elle était de 63 ans dans le groupe LAC. Le pourcentage des patients âgés de 60 ans ou plus dans les 2 groupes OC et LAC était respectivement de 63% et de 60%.Le sex-ratio était de 1,3 pour l’ensemble de l’effectif. Il était de 1,1 dans le groupeOC et de 1,5 dans le groupe LAC. Il n’yavait pas de différence significative entre les 2 groupes en terme de caractéristiques épidémiologiques. Les gestes opératoires intéressant le colon gauche représentaient 47 cas (72% de l’ensembledes interventions).La résection segmentaire basse était l’intervention la plus fréquemment réalisée soit dans 32%. Lestypes d’intervention dans les 2 groupes OC et LAC sont résumés dans le (Tableau 1). Il n’y avait pas de différence significative entre les 2 groupes en terme de gestes opératoiresréalisés (p= 0,940). La taille moyenne des tumeurs était de 5,3 cm (1→9) pour l’ensemble de l’effectif. Elle était de 5,7 cm (2→8) dans le groupe OC et de 5 cm (1→9) dans le groupe LAC. La différence n’était pas significative.

**Tableau 1:** Répartition des patients selon le type d’intervention dans les 2 groupes

Type d’intervention	Groupe OC	Groupe LAC	Total
Hémi-colectomie droite	10 (29%)	8 (27%)	18 (28%)
Hémi-colectomie gauche	8 (23%)	7 (23%)	15 (23%)
Résection segmentaire haute	5 (14%)	3 (10%)	8 (12%)
Résection segmentaire basse	11 (31%)	10 (33%)	21 (32%)
Hémi-colectomie gauche élargie au transverse	1 (3%)	2 (7%)	3 (5%)
**Total**	35 (100%)	30 (100%)	(100%)

OC : colectomie par voie ouverte ; LAC : colectomie laparoscopique

### Comparaison des résultats à court et à long terme

**Figure 1:**
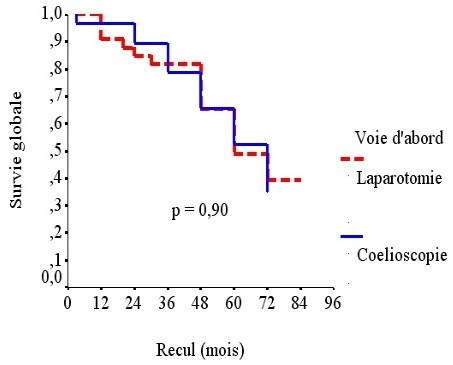
Survie globale tous stades confondus

**Figure 2:**
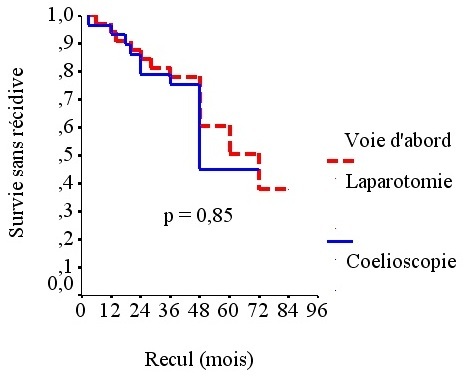
Survie sans récidive pour tous les stades confondus

Il n’avait pas été constaté de différence significative entre les deux groupes en termes de séjour en milieu de réanimation, douleurs postopératoires, reprise du transit, séjour hospitalier, morbidité et mortalité précoces ni en termes de complications tardives. Le taux de récidive était de 32% pour l’ensemble de l’effectif. Il était de 31% dans le groupe LAC et de 33% dans le groupe OC. La survie globale à 5 ans, tous stades confondus, était de 68%. Elle était de 69% dans le groupe LAC et de 67% dans le groupe OC (Figure 1). La différence n’était pas significative entre les 2 groupes. La survie sans récidive toutstade confondu était de 68%. Elle était de 69% dans le groupe LAC et de 67% dans le groupe OC (Figure 2). La différence n’était pas significative entre les 2 groupes (p= 0,85). Concernant la sécurité oncologique, toutes les limites carcinologiques étaient saines avec des marges de résection d’au moins 5 cm sur toutes les pièces de colectomies et ceci quelle que soit la voie d’abord. La moyenne du nombre de ganglions prélevés était de 13,2 (5→32) pour l’ensemble de l’effectif. Elle était de 12,9 (6→30) dans le groupe OC et de 13,6 (5→32) dans le groupe LAC sans qu’il y ait de différence significative entre les 2 groupes (p= 0,556). La synthèse des résultats de comparaison des résultats entre les deux groupes, avec un recul médian de 36 mois, est rapportée dans le (Tableau 2).

**Tableau 2:** Synthèse des résultats de comparaison

Critères de jugement		Groupe OC	Groupe LAC	p
**Données per-opératoires**	Temps opératoire* (en min)	136 (60à270)	222 (120à480)	<0,001
	Pertes sanguines* (en ml)	276 (100à700)	206 (100à1000)	0,246
	Recours à la transfusion sanguine (n)	5 (14%)	2 (7%)	0,265
	Taux de morbidité per-opératoire	0	.3%	0,276
**Suites opératoires**	Reprise du transit* (en j)	3,7 (1à7)	2,9 (1à7)	0,05
	Recours aux morphiniques (n)	20 (57%)	14 (47%)	0,276
	Séjour en milieu de réanimation* (en j)	4,5 (1à28)	2,8 (1à10)	0,155
	Séjour hospitalier* (en j)	8,2 (3à28)	6 (3à15)	0,096
	Taux de mortalité précoce	6%	3%	0,648
	Taux de morbidité précoce d’ordre médical	14%	10%	0,60
	Taux de morbidité précoce d’ordre chirurgical	23%	17%	0,70
	Taux des complications tardives	14%	7%	0,265
**Critères oncologiques**	Nombre de ganglions prélevés*	12,9 (6à30)	13,6 (5à32)	0,556
	Taux de mortalité liée au cancer	69%	60%	0,645
	Survie sans récidive pour tous stades confondus	67%	69%	0,82
	Survie sans récidive pour les stades I et II	82%	88%	0,96
	Survie sans récidive pour le stade III	50%	48%	0,70
	Survie globale pour tous les stades confondus	67%	69%	0,90
	Survie globale pour les stades I et II	76%	75%	0,62
	Survie globale pour le stade III	56%	62%	0,82

(n) : nombre de patients

* moyenne ;min : minute ; ml : millilitre ; OC : colectomie par voie ouverte ; LAC : colectomie laparoscopique

### Impact de la courbe d’apprentissage

**Figure 3:**
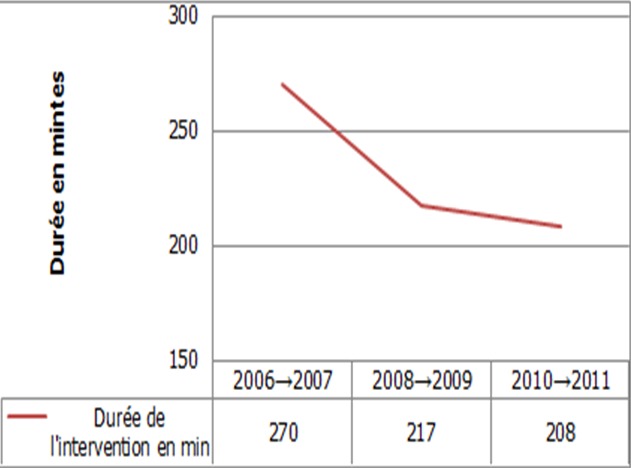
Temps opératoire moyen en fonction de l’année d’activité dans le groupe LAC

Le temps opératoire moyen était de 176 min (60 → 480) pour l’ensemble de l’effectif. Il était de 136 min (60→270) dans le groupe OC et de 222 min (120→480) dans le groupe LAC. Le temps opératoire était significativement plus long par laparoscopie par rapport à la laparotomie (p < 0,001). Le temps opératoire en fonction du type d’intervention et de la voie d’abord est résumé dans le (Tableau 3). Il faut noter que le temps opératoire en laparoscopie avait nettement diminué au fil des années (Figure 3). Le taux de conversion était de 33%. Il est passé de 67% au cours des 2 premières années de l’étude à 13% au cours des 2 dernières années. La conversion de la laparoscopie en laparotomie n’avait pas d’impact significatif ni sur les suites opératoires précoces, ni sur la survie globale et la survie sans récidive.

**Tableau 3:** Temps opératoire moyen en fonction du type d’intervention et de la voie d’abord

Type d’intervention	Groupe OC	Groupe LAC
Hémi-colectomie droite	141 (90 → 270) min	176 (150 → 210) min
Hémi-colectomie gauche	159 (120 → 240) min	287 (150 → 480) min
Résection segmentaire haute	180 (150→210) min	260 (180 → 360) min
Résection segmentaire basse	95 (60→120) min	210 (120 → 480) min
Hémi-colectomie gauche élargie au transverse	150 min	195 (50 → 240) min

min : minute ; OC : colectomie par voie ouverte ; LAC : colectomie laparoscopique

## Discussion

Nos résultats montrent que l’abord laparoscopique des ADK coliques non métastatiques est au moins équivalent à l’abord par laparotomie et ce à court et à long terme. En effet, nous n’avons pas noté de différence significative en terme de morbi-mortalité, de séjour hospitalier, de récidive, de survie globale ou sans récidive. La qualité d’exérèse attestée par les marges de résection et le nombre de ganglions prélevés était comparable entre les deux groupes. Nous avons également montré que la courbe d’apprentissage s’accompagnait d’un temps opéaratoire plus long pour la voie laparoscopique au début de la courbe. Elle s’accompagne également d’un taux de conversion important de 67% au cours des deux premières années. Le motif principal de cette conversion était les difficultés de dissection dans 60% des cas. Cette courbe d’apprentissage ne s’accompagnecependant pas d’une sur-morbimortlité opératoire et n’altère pas la survie globale ni la survie sans récidive. Les limites de cette étude sont essentiellement représentés parle caractère rétrospectif et monocentrique. La voie laparoscopique dans le traitement des adénocarcinomes coliques non métastatiques est faisable et reproductible. Elle procure une sécurité oncologique au moins équivalente à la laparotomie.La plupart des auteurs s’accordent sur le fait que la voie d’abord ne modifie pas le taux de morbidité ni de mortalité per-opératoire [2–5].

Nos résultats confirment cette idée largement présente dans la littérature. L’objectif de la chirurgie carcinologique est d’obtenir une exérèse en monobloc avec des marges de résection saines d’au moins 3 cm pour les tumeurs T1, d’au moins 5 cm pour les tumeurs T2 et d’au moins 7 cm pour les tumeurs infiltrantes (T3 et T4) [6]. Il n’a pas été rapporté de différence significative concernant les marges de résections carcinologiques entre laparoscopie et laparotomie dans les différentes séries [7–10]. L’étude de Sun affiche même une différence significative en faveur de la laparoscopie en termes de longueur de la marge de résection proximale [11]. L’étendue de l’exérèse ganglionnaire dans la chirurgie du cancer du colon définit la qualité du curage [12]. La plupart des auteurs s’accordent sur le fait que le choix de la voie d’abord n’influence pas la qualité du curage ganglionnaire. Dans les études comparatives et randomisées, la moyenne du nombre des ganglions prélevés variait de 10 à 26 en laparotomie et de 10 à 23 en laparoscopie sans différence significative entre les 2 voies d’abord [2, 4, 5, 8, 9, 13, 14]. Dans notre étude, la sécurité carcinologique est attestée par les marges de résections qui étaient comparables entre les deux groupes. Le nombre de ganglions prélevés était de 12,9 en moyenne en laparotomie Vs 13,6 en moyenne en laparoscopie. L’étude de Braga a étudié les complications tardives en fonction la voie d’abord. Les résultats avaient montré l’équivalence de la laparoscopie et de la chirurgie ouverte en terme de complications tardives. La méta-analyse de Kuhry avait inclus 12 études prospectives et randomisées et 3.346 patients. Elle avait conclu à l’absence de différence significative entre les 2 voies d’abord en termes d’éventration (p= 0,32) et de ré-intervention pour occlusion sur brides postopératoires (p= 0,30) [15]. Nous ne retrouvons pas de différence significative entre les deux groupes en termes de complications tardives.

Aucune différence statistique en terme de survie globale n’avait été retrouvée dans la plupart des études randomisées et les méta-analyses comparant les colectomies laparoscopiques aux colectomies par laparotomie dans le traitement des cancers du colon [3, 4, 15–22].Plusieurs études avaient même montré l’équivalence de la laparoscopie et de la laparotomie en terme de survie sans récidive [3, 4, 15–22]. Notre étude confirme ces données puisqu’il n’y avait de différence significative entre les deux groupes en termes de récidive, survie globale et survie sans récidive. La courbe d’apprentissage ne doit pas constituer un frein à l’utilisation de la laparoscopie en particulier dans les cancers coliques non métastatiques. Une équipe asiatique a étudié l’impact de la courbe d’apprentissage dans un hôpital régional et a montré que cette voie d’abord était parfaitement sure et reproductible [23]. Dans notre série, la courbe d’apprentissage a eu un impact sur le temps opératoire sans pour autant influencer la morbi-mortalité, la récidive ou la survie.

## Conclusion

La voie d’abord laparoscopique est une voie d’abord ayant des résultats à court et à long terme au moins équivalents à la laparotomie. La courbe d’apprentissage représentant un « passage obligé » n’a pas d’impact négatif sur les résultats du traitement laparoscopique des cancers coliques non métastatiques. L’abord laparoscopique doit être développé dans le monde méditaranéo-africain même s’il reste dépendant d’un plateau technique adéquat.

### Etat des connaissances actuelle sur le sujet

- L’abord laparoscopique est validé dans le traitement des cancers du colon non métatstatiquesdasn les pays occidentaux. Il donnerait les mêmes résultats carcinologiques que l’abord par laparotomie.

### Contribution de notre étude à la connaissance

- La validation de la chirurgie laparoscopique pour cancer du colon dans un centre de chirurgie viscérale d’Afrique du nord. Montrer que nos résultats sont comparables aux séries occidentales. Insister sur le fait que la courbe d’apprentissage ne doit pas représenter un frein au développement de cette voie d’abord. Cette courbe d’apprentissage n’a pas à proprement parler de résultats néfastes.
